# ﻿Three new species of jumping spiders (Araneae, Salticidae) from Hunan, China

**DOI:** 10.3897/zookeys.1204.122887

**Published:** 2024-06-07

**Authors:** Song-Lin Li, Ping Liu, Xian-Jin Peng

**Affiliations:** 1 College of Life Sciences, Hunan Normal University, Changsha, Hunan 410081, China Hunan Normal University Changsha China

**Keywords:** Bamian Mountain, barcoding gene, COI, taxonomy

## Abstract

Three new species of the genera *Thiania* C. L. Koch, 1846 and *Yaginumaella* Prószyński, 1979 are described and named as *T.bamian***sp. nov.** (♂♀), *T.flacata***sp. nov.** (♀) and *Y.curvata***sp. nov.** (♂♀), from Hunan Province, China. Detailed descriptions, photos of somatic features and copulatory organs, as well as a distribution map are provided. Nucleotide data for the barcoding gene, cytochrome c oxidase subunit I (COI) of *T.bamian***sp. nov.** (♂♀) and *Y.curvata***sp. nov.** (♀) are provided.

## ﻿Introduction

*Thiania* C. L. Koch, 1846 is a well-known genus of the tribe Euophryini. It currently comprises 25 species mainly distributed in Asia, of which eight species are known from China (WSC 2024). Species belonging to *Thiania* can be recognized by the rectangular flattened carapace and robust leg I (Prószyński 2009).

*Yaginumaella* Prószyński, 1979 is currently placed in the tribe Plexippini according to molecular analysis (Maddison 2015). There are 21 species recorded from China (Zhu et al. 2005; Peng et al. 2008; Shao et al. 2014; Liu et al. 2016; Li et al. 2018; Peng 2020; Wang et al. 2023; Wang et al. 2024).

While examining specimens collected from Bamian Mountain, two new species of *Thiania* and one new species of *Yaginumaella* were recognized and are described here.

## ﻿Materials and methods

Specimens are stored in 100% ethanol. Vulvae were cleaned with trypsin solution before examination and photography. Left male palps were dissected and used for description and color photos. Specimens were examined and measured with a Leica M205C stereomicroscope. Photos were taken with a digital camera Kuy Nice E3IS PM mounted on an Olympus BX53. Compound focus-stacked images were generated using Helicon Focus v. 7.6.1 and then adjusted in Adobe Photoshop 2020. The map was created by ArcMap v. 10.8. All measurements are given in millimeters (mm). Leg measurements are given in the following order: total length (femur, patella + tibia, metatarsus, tarsus). Genomic DNA was extracted from four legs of each specimen using an Animal Genomic DNA Isolation Kit (Tiangen Biotech, Beijing, China), and the universal primer pair LCO1490/HCO2198 was used for amplification of the COI gene (Folmer et al. 1994). The PCR products were sent to Tsingke Biotechnology Co., Ltd (Changsha, China) for purification and sequencing. The obtained sequences were aligned using Geneious Prime v. 9.0.2. The COI GenBank accession numbers of *T.bamian* sp. nov. (♂♀) and *Y.curvata* sp. nov. (♀) are also provided.

Specimens are deposited in the College of Life Sciences, Hunan Normal University (**HUNNU**) in Changsha, China. Abbreviations used are as follows: **AER** anterior eye row, **ALE** anterior lateral eye, **AME** anterior median eye, **CD** copulatory duct, **CO** copulatory opening, **E** embolus, **ED** embolic disc, **EFL** length of eye field, **EW** epigynal window, **FD** fertilization duct, **TL** tegular lobe, **MOA** median ocular area, **P** pocket, **PER** posterior eye row, **PLE** posterior lateral eye, **PME** posterior median eye, **RTA** retrolateral tibial apophysis, **S** spermatheca, **SD** sperm duct.

## ﻿Taxonomy

### ﻿Family Salticidae Blackwall,1841

#### ﻿Genus Thiania

Taxon classificationAnimaliaAraneaeSalticidae

C. L. Koch, 1846

20E460AF-3121-5F5A-B8CA-15DD9D30745D

##### Type species.

*Thianiapulcherrima* C. L. Koch, 1846.

#### ﻿ Thiania bamian sp. nov.

Taxon classificationAnimaliaAraneaeSalticidae

30B5949F-FFDF-5E95-967A-ECA54093C28F

https://zoobank.org/77A1258A-56A1-4564-81D8-3BE0D8DEB134

Figs 1 , 2 , 6

##### Type material.

***Holotype*** ♂ (HNU-BMS-1905), China, Hunan Prov., Chenzhou City, Guidong Co., Bamian Mountain National Nature Reserve, 25.975210°N, 113.702865°E, 1081 m, 18 Sept. 2019, Cheng Wang, Bo Lü and Xuan-Wei Zhou leg.; ***paratypes***: 1♀ (HNU-BMS-1903), China, Hunan Prov., Chenzhou City, Guidong Co., Bamian Mountain National Nature Reserve, 26.001944°N, 113.710675°E, 1678 m, 16 Sept. 2019, Cheng Wang, Bo Lü and Xuan-Wei Zhou leg.; 1♀ (HNU-BMS-2201), China, Hunan Prov., Chenzhou City, Guidong Co., Bamian Mountain National Nature Reserve, 25.978498°N, 113.713744°E, 1025 m, 18 Aug. 2022, Song-Lin Li, Peng Yong, Li-Fen Li, Yu-Chen Zhou, Zi-Yue Liu leg.

##### Etymology.

The specific epithet is derived from the type locality Bamian Mountain National Nature Reserve, noun.

##### Diagnosis.

**Figure 1.**
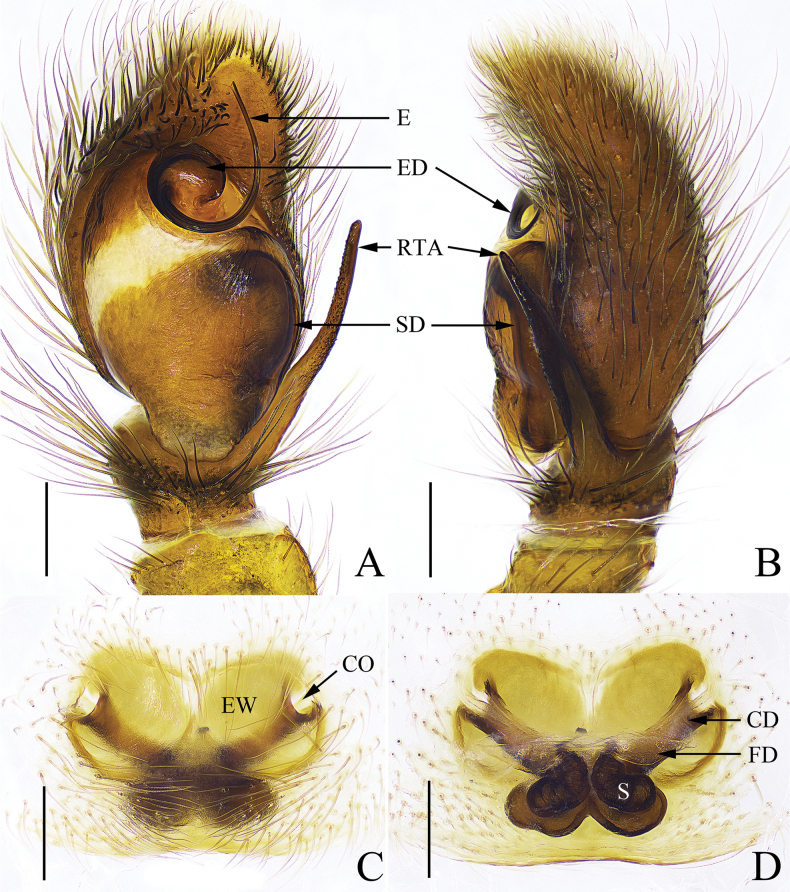
*Thianiabamian* sp. nov. **A** left palp, ventral view **B** ditto, retrolateral view **C** epigyne, ventral view **D** vulva, dorsal view. Scale bars: 0.2 mm.

The male of this new species is similar to that of *Thianialongapophysis* Yu & Zhang, 2022 (Yu and Zhang 2022: figs 7A–D, 8A, B) in the shape of palpal bulb, sperm duct and embolus, but can be distinguished by: 1) the angle between RTA and cymbium (Fig. 1A) smaller than that angle in *T.longapophysis* (fig. 8A); and 2) the distal end of RTA bar-shaped (Fig. 1A), while barb-shaped in *T.longapophysis* (fig. 8A). The female of this new species is similar to that of *Thianialuteobrachialis* Schenkel, 1963 (Peng 2020: fig. 352a–c) in the shape of the epigynal window and the location of copulatory openings, but can be distinguished by the following characters: 1) proximal portion of copulatory ducts straight and V-shaped (Fig. 1D), while curved and U-shaped in *T.luteobrachialis* (fig. 352c); and 2) spermathecae overlapping with copulatory ducts (Fig. 1D), while not overlapping with copulatory ducts in *T.luteobrachialis* (fig. 352c).

##### Description.

**Figure 2.**
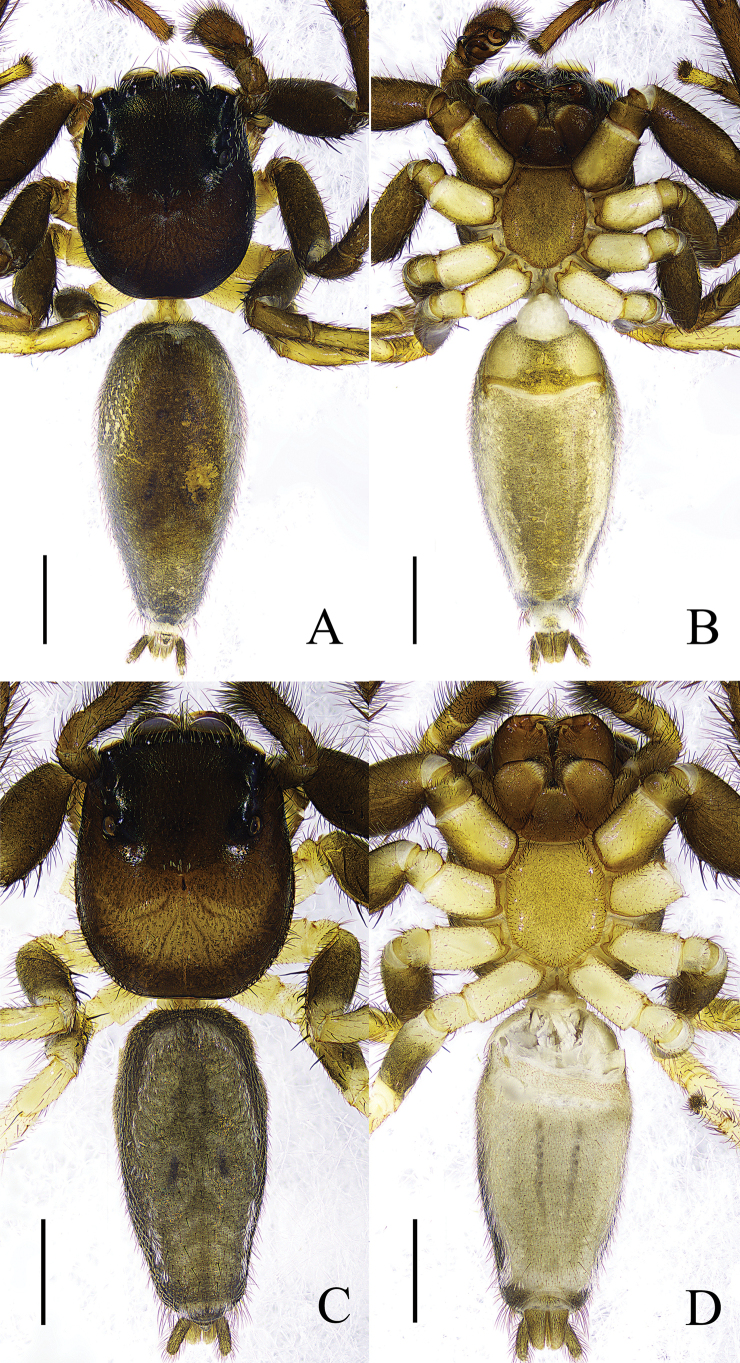
*Thianiabamian* sp. nov. **A** male holotype, habitus, dorsal view **B** ditto, ventral view **C** female paratype, habitus, dorsal view **D** ditto, ventral view. Scale bars: 1 mm.

**Male** (holotype) (Fig. 2A, B). Total length 6.02; carapace 2.39 long, 2.03 wide; abdomen 3.45 long, 1.73 wide. Clypeus 0.24 high. Carapace dark brown, eye field covered with white setae. Fovea longitudinal, radial grooves distinct, cervical grooves indistinct. Eye sizes and interdistances: AME 0.50, ALE 0.29, PME 0.04, PLE 0.15, AER 1.64, PER 1.48, EFL 1.14. Chelicerae dark brown, promargin with one bicuspid tooth, retromargin with one tooth. Endites and labium brown, distal end pale yellow, with dark setae. Sternum yellow brown. Leg pale yellow to brown. Measurements of legs: I 7.27 (2.01, 3.03, 1.51, 0.72), II 5.04 (1.65, 1.76, 1.03, 0.60, 5.04), III 4.91 (1.52, 1.68, 1.22, 0.49), IV 4.82 (1.42, 1.80, 1.07, 0.53). Leg formula: 1234. Abdomen dorsum brown, with lighter edges; venter light yellow, with a wide light brown longitudinal band in the center.

Palp (Fig. 1A, B). Embolus long and thin, embolic disc distinct; retrolateral tibial apophysis long and thin, distal portion covered with many small granules, terminal end reached the antero-median portion of palpal bulb in retrolateral view; sperm duct obvious.

**Female** (paratype) (Fig. 2C, D). Total length 5.49; carapace 2.58 long, 2.11 wide; abdomen 2.86 long, 1.51 wide. Clypeus 0.32 high. Carapace yellow brown, with dark eye file and margins. Eye sizes and interdistances: AME 0.44, ALE 0.25, PME 0.05, PLE 0.20, AER 1.56, PER 1.52, EFL 1.14. Leg pale yellow to brown. Measurements of legs: I 5.47 (1.59, 2.29, 0.95, 0.64), II 4.44 (1.40, 1.72, 0.78, 0.54), III 4.51 (1.43, 1.60, 1.02, 0.46), IV 4.71 (1.42, 1.77, 1.09, 0.43). Leg formula: 1432. Abdomen dorsum brown, edges darker and with white hair, median portion with one pair of dark patches, posterior portion with four dark triangular patterns; venter pale yellow, median portion with one pair of gray longitudinal lines. Color paler than that in male.

Epigyne (Fig. 1C, D). Epigynal window circular. Copulatory openings oval, located on both sides of the epigynal window. Copulatory ducts with straight original portion and coiled terminal portion. Spermathecae shoe-shaped, slightly narrower than the copulatory ducts and overlapping with terminal portion of copulatory ducts.

##### Distribution.

Known only from the type locality (Fig. 6).

##### GenBank accession number.

Holotype (HNU-BMS-1905): PP786559; paratype ((HNU-BMS-2201): PP786560.

#### ﻿ Thiania flacata sp. nov.

Taxon classificationAnimaliaAraneaeSalticidae

EC2B6D7B-1F84-5E06-9866-C7ADB158AE2A

https://zoobank.org/A4578588-C911-4797-A06E-2CD405B48E63

Figs 3 , 6

##### Type material.

***Holotype*** ♀ (HNU-BMS-1905), China, Hunan Prov., Chenzhou City, Guidong Co., Bamian Mountain National Nature Reserve, 25.975210°N, 113.702865°E, 1081 m, 18 Sept. 2019, Cheng Wang, Bo Lü and Xuan-Wei Zhou leg.

##### Etymology.

The specific epithet is derived from the Latin “*falcata*” (falx-shaped), referring to the falx-shaped copulatory ducts, adjective.

##### Diagnosis.

This new species can be distinguished from any other congeneric species by the vaulted copulatory openings.

##### Description.

**Figure 3.**
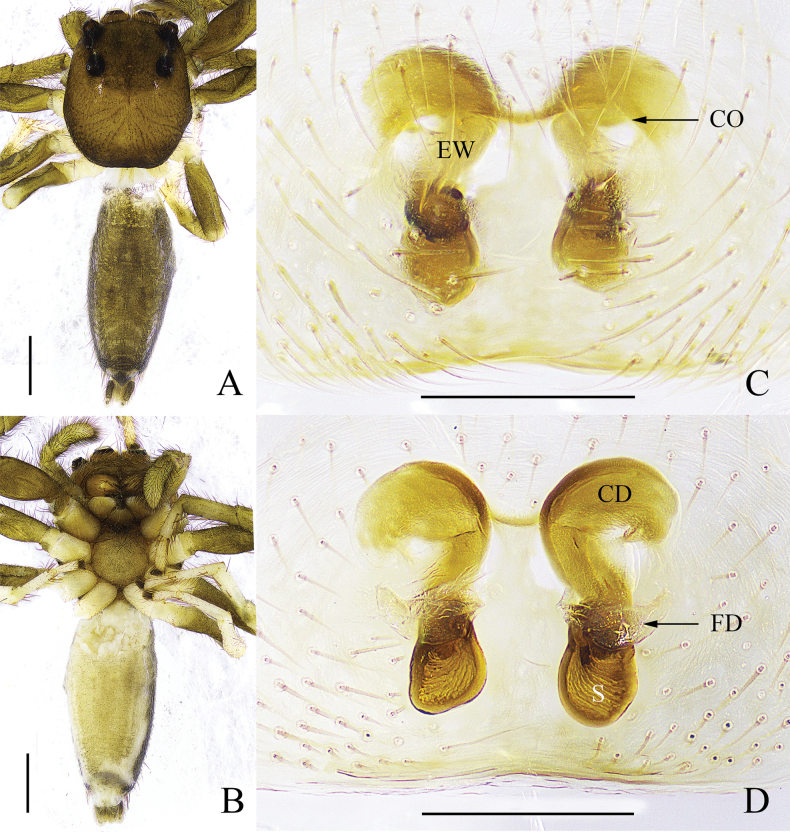
*Thianiaflacata* sp. nov. **A** female paratype, habitus, dorsal view **B** ditto, ventral view **C** epigyne, ventral view **D** vulva, dorsal view. Scale bars: 1 mm (**A, B**); 0.2 mm (**C, D**).

**Female** (holotype) (Fig. 3A, B). Total length 6.04; carapace 2.58 long, 2.24 wide; abdomen 3.37 long, 1.52 wide. Clypeus 0.12 high. Carapace yellow brown, with brown eye file and margins. Fovea longitudinal, radial and cervical grooves distinct. Eye sizes and interdistances: AME 0.51, ALE 0.27, PME 0.06, PLE 0.14, AER 1.62, PER 1.50, EFL 1.07. Chelicerae yellow brown, promargin with two teeth, retromargin with one tooth. Endites and labium yellow brown, distal end pale yellow with dark setae. Sternum yellow brown. Leg pale yellow to brown. Measurements of legs: I 6.33 (1.65, 2.65, 1.35, 0.68), II 4.58 (1.33, 1.83, 0.77, 0.65), III 4.61 (1.38, 1.67, 1.08, 0.48), IV 4.61 (1.40, 1.79, 0.99, 0.43). Leg formula: 1342. Abdomen dorsum brown, median portion with one pair of dark patches, posterior portion with three transverse light herringbone patterns, edges with white setae; venter light yellow, with a wide light brown longitudinal band in the center.

Epigyne (Fig. 3C, D). Epigynal window located medially on epigyne. Copulatory openings vaulted, located at the upper margin of the epigynal window. Copulatory ducts falx-shaped, original portion thicker. Spermathecae oval, slightly wider than the copulatory ducts.

**Male.** Unknown.

##### Distribution.

Known only from the type locality (Fig. 6).

#### ﻿Genus Yaginumaella

Taxon classificationAnimaliaAraneaeSalticidae

Prószyński, 1979

19F4AB9E-3028-563F-BAF4-4E9ACA99BFCA

##### Type species.

*Yaginumaellastriatipes* Grube, 1861.

##### Remarks.

The genus *Yaginumaella* is currently placed in the Plexippini tribe according to molecular analysis, together with the genus *Ptocasius* (Maddison 2015). *Yaginumaella* closely resembles *Ptocasius* (Logunov and Jäger 2015), especially in females (Li et al. 2018). However, there are clear differences in the type species of *Yaginumaella* and *Ptocasius* (Prószyński 2017). Patoleta et al. (2020), transferred 37 species of *Yaginumaella* to the genus *Ptocasius* only based on the similarity of genitalic structures. But, based on the characteristics shown in literature illustrations of species, these two genera can be distinguished by the following characters: 1) carapace with light longitudinal stripes in *Yaginumaella*, while usually with transverse stripes in *Ptocasius*; and 2) palpal bulb enlarged, with tegular lobe in *Yaginumaella*, while oblate, without tegular lobe in *Ptocasius* (Li et al. 2018). Therefore, according to the above characteristics, *Y.curvata* sp. nov. is described as a member of the genus *Yaginumaella*. In addition, based on the close collecting locations and genital characteristics of males and females, we tentatively identify them as the same species.

#### ﻿ Yaginumaella curvata sp. nov.

Taxon classificationAnimaliaAraneaeSalticidae

3759E5DE-DE7F-59F2-8DF6-906FED99A66B

https://zoobank.org/C54A80DD-0FBE-42A5-9382-6BD123C9F2B5

Figs 4 , 5 , 6

##### Type material.

***Holotype*** ♂ (HNU-BMS-1901), China, Hunan Prov., Chenzhou City, Guidong Co., Bamian Mountain National Nature Reserve, 25.975914°N, 113.708825°E, 1001 m, 15 Sept. 2019, Cheng Wang, Bo Lü and Xuan-Wei Zhou leg.; ***paratypes***: ♀ (HNU-BMS-2202), China, Hunan Prov., Chenzhou City, Guidong Co., Bamian Mountain National Nature Reserve, 25.975568°N, 113.705383°E, 1143 m, 19 Aug. 2022, Song-Lin Li, Peng Yong, Li-Fen Li, Yu-Chen Zhou, Zi-Yue Liu leg.; 2♀ (HNU-BMS-2205), China, Hunan Prov., Chenzhou City, Guidong Co., Bamian Mountain National Nature Reserve, 25.986542°N, 113.705841°E, 1250 m, 22 Aug. 2022, Song-Lin Li, Peng Yong, Li-Fen Li, Yu-Chen Zhou, Zi-Yue Liu leg.

##### Etymology.

The specific epithet is derived from the Latin “*curvata*” (curved), referring to the curved retrolateral tibial apophysis, adjective.

##### Diagnosis.

**Figure 4.**
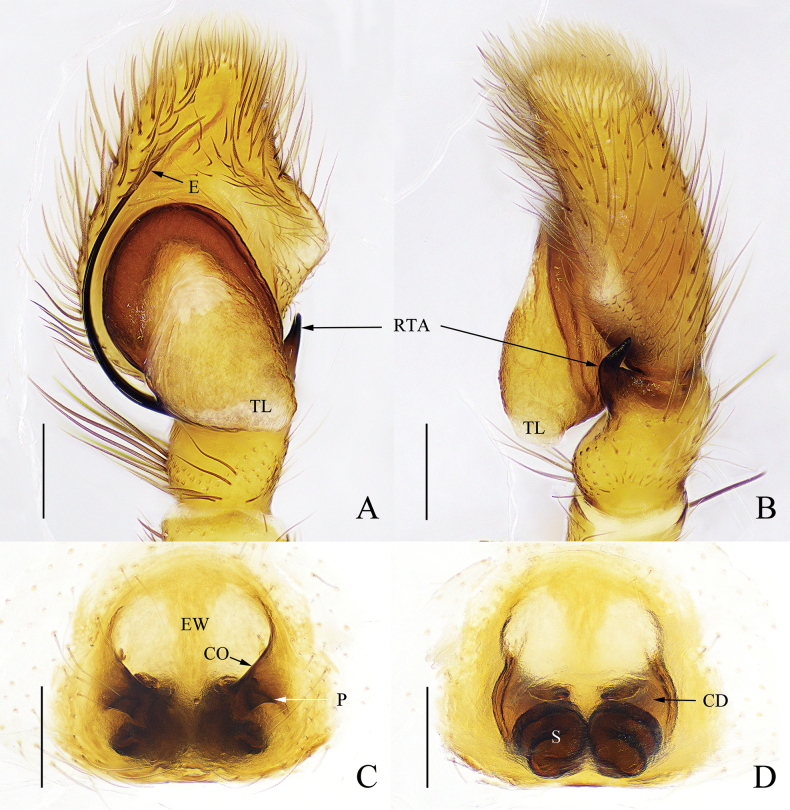
*Yaginumaellacurvata* sp. nov. **A** left palp, ventral view **B** ditto, retrolateral view **C** epigyne, ventral view **D** vulva, dorsal view. Scale bars: 0.2 mm.

The male of this new species is similar to that of *Yaginumaellabulbosa* Peng, Tang & Li, 2008 (Peng et al. 2008: figs 26–28) in habitus and the curved RTA, but can be distinguished by: 1) cymbium longer than wide (Fig. 4A), while wider than long in *Y.bulbosa* (fig. 27); 2) length of RTA is about 1/3 of the palpal bulb (Fig. 4B), while about 1/2 of the palpal bulb in *Y.bulbosa* (fig. 28); 3) RTA only extended to the basal 1/6 position of cymbium in retrolateral view (Fig. 4B), while to the basal 1/2 position of cymbium in retrolateral view in *Y.bulbosa* (fig. 28); and 4) embolus originates at about 7:00 o’clock position (Fig. 4A), while originates at about 9:00 o’clock position in *Y.bulbosa* (fig. 27). The female of this new species is similar to that of *Yaginumaellalushiensis* Zhang & Zhu, 2007 (Zhu and Zhang 2011: fig. 384A, D, E) in the short and stout copulatory ducts and the shape of spermathecae, but can be distinguished by the following characters: 1) distance of copulatory openings as wide as the vulva (Fig. 4C), while about 1/3 width of the vulva in *Y.lushiensis* (fig. 384D); 2) epigynal pockets located at the median portion of epigyne (Fig. 4C), while located on the anterior portion in *Y.lushiensis* (fig. 384D); and 3) fertilisation duct about transverse (Fig. 4D), while oblique in *Y.lushiensis* (fig. 384E).

##### Description.

**Figure 5.**
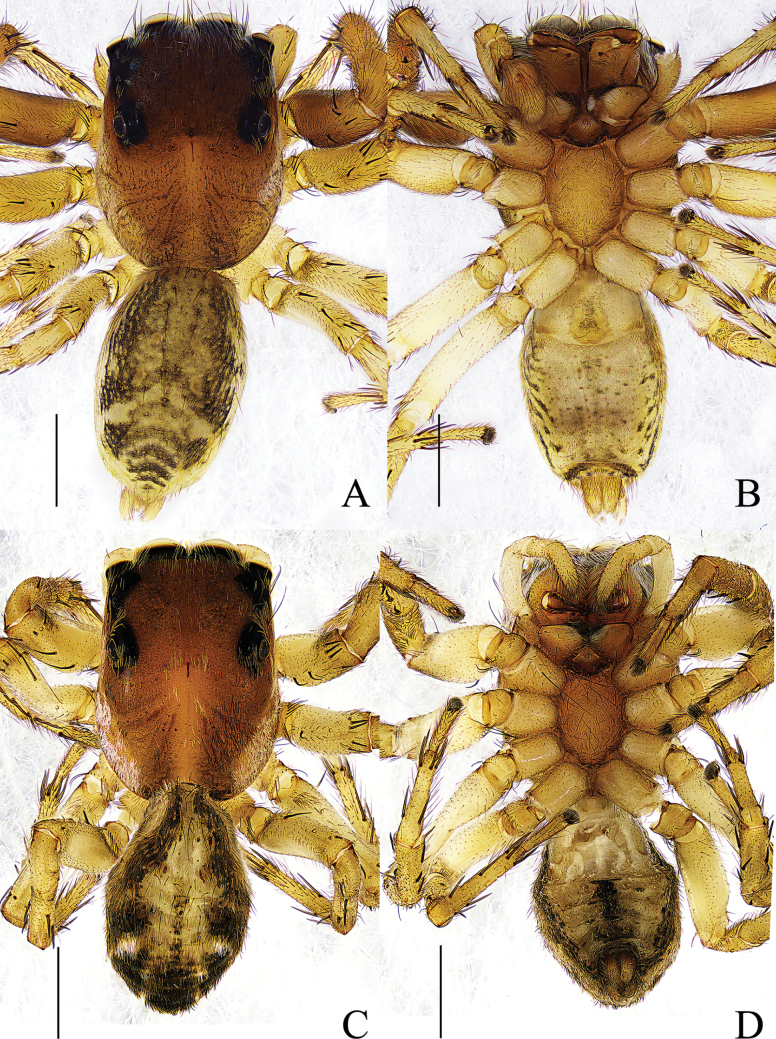
*Yaginumaellacurvata* sp. nov. **A** male holotype, habitus, dorsal view **B** ditto, ventral view **C** female paratype, habitus, dorsal view **D** ditto, ventral view. Scale bars: 1 mm.

**Male** (holotype) (Fig. 5A, B). Total length 5.14; carapace 2.60 long, 2.06 wide; abdomen 2.52 long, 1.59 wide. Carapace brown, with three longitudinal yellow stripes on the median and lateral margins, eye field and lateral margins covered with white setae. Fovea longitudinal, cervical and radial grooves distinct. Eye sizes and interdistances: AME 0.49, ALE 0.28, PME 0.08, PLE 0.21, AER 1.75, PER 1.65, EFL 0.78. Chelicerae light brown, promargin with two teeth, and retromargin with one tooth. Endites and labium light brown, distal end pale yellow. Sternum pale yellow. Legs yellow except for femora, patellae and tibiae of leg I brown. Measurements of legs: I 5.99 (1.76, 2.38, 1.17, 0.68), II 4.89 (1.59, 1.91, 0.67, 0.72), III 5.39 (1.70, 1.94, 1.14, 0.61), IV 5.50 (1.76, 1.79, 1.32, 0.63). Leg formula: 1432. Abdomen oval, dorsum with sparse long black hair, anterior margin black, the posterior median with two gray herringbone patterns and four chevrons; venter light yellow, with black maculation.

Palp (Fig. 4A, B). Embolus long and thin, originates at about 7:00 o’clock position; tegular lobe folds to retrolateral side; retrolateral tibial apophysis curved towards dorsal side at right angle from the middle.

**Female.** (paratype) (Fig. 5C, D). Total length 4.94; carapace 2.74 long, 2.04 wide; abdomen 2.54 long, 1.63 wide. Eye sizes and interdistances: AME 0.41, ALE 0.27, PME 0.11, PLE 0.23, AER 1.81, PER 1.79, EFL 0.72. Chelicerae promargin with two teeth, retromargin with one tooth. Legs pale yellow. Measurements of legs: I 4.79 (1.63, 1.80, 0.76, 0.60), II 4.44 (1.71, 1.49, 0.69, 0.55), III 5.49 (1.82, 1.94, 1.08, 0.65), IV 5.23 (1.63, 1.87, 1.03, 0.70). Leg formula: 3412. Abdomen oval, dorsum black, with symmetric lighter yellowish central area, posterior portion covered with dense black and white long hairs; venter light yellow, with three longitudinal black stripes. Color darker than that in male.

Epigyne (Fig. 4C, D). Epigynal window oval, located at anterior portion of epigyne. Copulatory openings slit-shaped, located at the lower lateral margin of the epigynal window. Copulatory ducts short and stout. Spermathecae tubular and intertwined.

##### Distribution.

**Figure 6.**
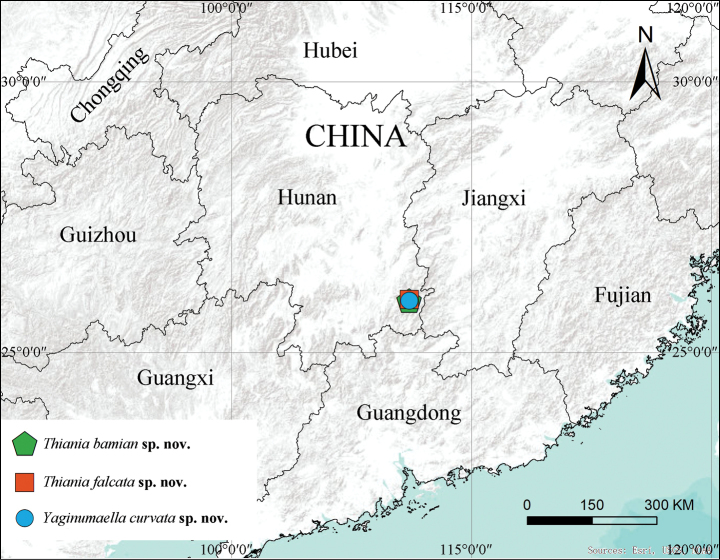
Collection localities of *Thianiabamian* sp. nov., *Thianiaflacata* sp. nov. and *Yaginumaellacurvata* sp. nov.

Known only from the type locality (Fig. 6).

##### GenBank accession number.

Paratype ((HNU-BMS-2205): PP786561.

## Supplementary Material

XML Treatment for Thiania

XML Treatment for Thiania bamian

XML Treatment for Thiania flacata

XML Treatment for Yaginumaella

XML Treatment for Yaginumaella curvata

## ﻿Additional information

### Author ORCIDs

Song-Lin Li https://orcid.org/0000-0002-1127-0781

Ping Liu https://orcid.org/0000-0002-4959-2735

Xian-Jin Peng https://orcid.org/0000-0002-2614-3910

### Data availability

All of the data that support the findings of this study are available in the main text.
